# *In vitro* NTPase activity of highly purified Pdr5, a major yeast ABC multidrug transporter

**DOI:** 10.1038/s41598-019-44327-8

**Published:** 2019-05-23

**Authors:** Manuel Wagner, Sander H. J. Smits, Lutz Schmitt

**Affiliations:** Institute of Biochemistry, Heinrich-Heine-Universität Düsseldorf, Universitätsstraße 1, 40225 Düsseldorf, Germany

**Keywords:** Membrane proteins, Biochemical assays

## Abstract

The ABC transporter Pdr5 of *S*. *cerevisiae* is a key player of the PDR network that works as a first line of defense against a wide range of xenobiotic compounds. As the first discovered member of the family of asymmetric PDR ABC transporters, extensive studies have been carried out to elucidate the molecular mechanism of drug efflux and the details of the catalytic cycle. Pdr5 turned out to be an excellent model system to study functional and structural characteristics of asymmetric, uncoupled ABC transporters. However, to date studies have been limited to *in vivo* or plasma membrane systems, as it was not possible to isolate Pdr5 in a functional state. Here, we describe the solubilization and purification of Pdr5 to homogeneity in a functional state as confirmed by *in vitro* assays. The ATPase deficient Pdr5 E1036Q mutant was used as a control and proves that detergent-purified wild-type Pdr5 is functional resembling in its activity the one in its physiological environment. Finally, we show that the isolated active Pdr5 is monomeric in solution. Taken together, our results described in this study will enable a variety of functional investigations on Pdr5 required to determine molecular mechanism of this asymmetric ABC transporter.

## Introduction

ATP-binding cassette (ABC) transporters are ubiquitous, primary active membrane proteins that are found in all kingdoms of life^1^. In general, they can be divided into two classes, depending on the direction of transport: ABC importers (including ECF transporters)^2^ and exporters^3^. A functional unit of an ABC transporter consists of two nucleotide-binding domains (NBDs) that bind and hydrolyze ATP to energize the transport cycle, and two transmembrane domains (TMDs), which form the translocation pathway across the membrane^1^. Overexpression of ABC transporters that export toxic compounds is part of a phenomenon known as multidrug resistance and represents a main obstacle in chemo-therapeutic cancer treatment as well as bacterial infections^4,5^. In fungi and plants the overexpression of these drug efflux pumps is part of the pleiotropic drug resistance (PDR) network^6^. All PDR ABC transporters are, with the exception of Adp1, full-size transporter and possess a reverse topology of (NBD-TMD)_2_^7,8^. The ABC transporter Pdr5 of *Saccharomyces cerevisiae* has been established as a model for fungal PDR proteins and studied for more than 25 years^9^. It confers resistance towards a broad range of structurally and functionally different substrates including azoles, ionophores, antibiotics and many others^10,11^. However, the nature of the physiological substrate(s) is not known. The expression of PDR ABC transporters is regulated through a complex regulatory network of transcription factors, of which the zinc finger regulators Pdr1 and Pdr3 are mainly responsible for Pdr5 regulation^12^. A mutation in Pdr1 (*pdr1–3*) is used for constitutive overexpression of the transporter^13^, which can also be used to overexpress other ABC transporter using this system^14^.

ABC transporters all share conserved motifs within their NBDs, namely the Walker A and B, the signature motif (or C-loop) as well as the D- and H-loop^15^. Pdr5, however, features substitutions of key residues in each motif, except of the D-loops, in one of its nucleotide binding sites (NBSs), which renders this NBS ATPase deficient, also known as degenerated. Therefore, Pdr5 belongs to the family of asymmetric ABC transporters amongst CFTR, MRP1, ABCG5/ABCG8 and others^16–18^. It remains elusive what the exact physiological function of the degenerated NBS is, but it is obvious that there is an essential function as restoring the canonical motifs leads to inactive Pdr5^19^. There are however, indications that the degenerated NBS is involved in the interdomain crosstalk between the NBDs and the TMDs. Single mutations within the deviant NBS did not impact the overall ATPase activity of the transporter while they severely affected the transport functionality^19,20^. The crystal structure of TM287/288^21^ nicely demonstrated the consequence of one catalytic and one non-canonical site, e. g. only a bound nucleotide (ATP) at the non-canonical site. However, one has to stress that a gradient of asymmetry exists in asymmetric ABC transporters. The number of mutations in the catalytically relevant motifs resulting in the inactivation of the corresponding nucleotide-binding site ranges from a few (e.g. two mutations in ABCG5/ABCG8^16^ or three in CFTR^22^ and TM287/288^21^) to all motifs forming the NBS (Pdr5 and its homologue in *Candida albicans* Cdr1^23^). This obviously raises the questions whether or not a relation between number of disrupted motifs and the molecular mechanism of substrate transport exists.

Pdr5 from *Saccharomyces cerevisiae* was the first identified member of the PDR subfamily of asymmetric ABC transporters^9^. Due to the medical significance of mammalian homologues and the agricultural importance of plant and other fungal homologues, the yeast PDR system serves as a unique model to investigate their molecular mechanisms.

Moreover, Pdr5 exhibits a high basal ATPase activity that, in contrast to other ABC transporters such as P-gp^24^, cannot be further stimulated in the presence of its substrates, uncoupling the ATPase activity from drug efflux^25^. Although there is a long history of studies related to Pdr5, it has not been accomplished to successfully purify the ABC transporter and to study it in detail in an isolated system, which is a prerequisite to fully understand the molecular mechanisms of drug binding and transport.

## Results

### Isolation and purification of Pdr5 in a functional form

**Figure 1:**
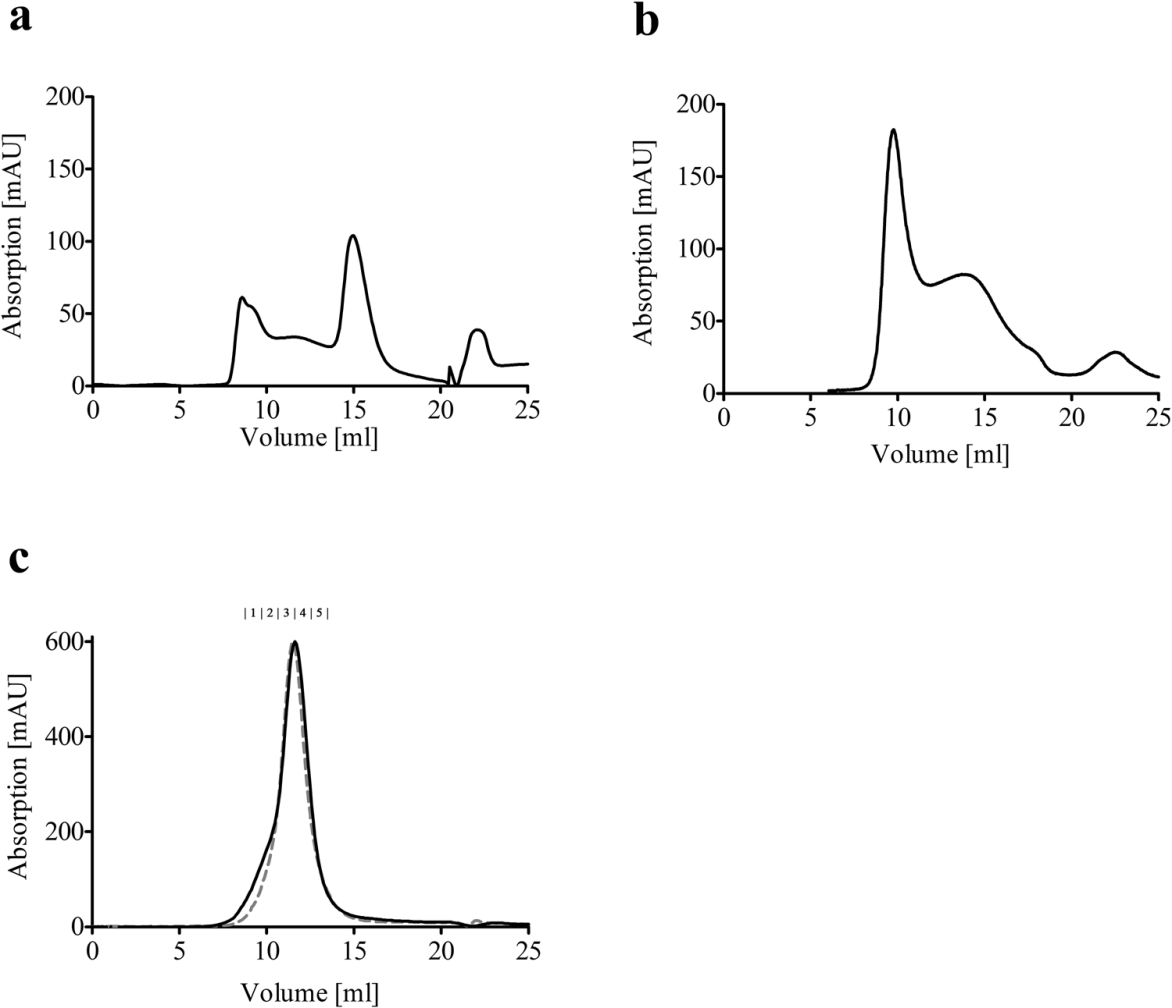
Size exclusion chromatograms of Pdr5 after affinity purification with different detergents. (**a**) SEC of Pdr5 purified with DDM. (**b**) SEC of Pdr5 purified with C_12_E_8_. (**c**) SEC of wild-type (black solid line) and E1036Q (gray dashed line) Pdr5 purified with trans-PCC-α-M. SEC was performed in buffer A (50 mM Tris-HCl pH 7.8, 50 mM NaCl, 10% glycerol and 0.05% DDM, 0.01% C_12_E_8_ or 0.003% trans-PCC-α-M respectively) on a Superdex 200 10/300 GL column (GE Healthcare). The concentration of Pdr5 was 2 mg/ml. The elution fractions that were analyzed by SDS-PAGE and Western blot (Fig. 2) are indicated above the chromatograms.

In order to establish the purification of Pdr5 in a functional state at high purity and yield, we screened 20 different detergents for protein solubilization. In the course of these experiments, it turned out that PCC-α-M was the most suitable detergent for solubilization as well as for subsequent affinity purification and size exclusion chromatography. Figure 1 shows three selected examples of size exclusion chromatograms of Pdr5 after affinity purification in the presence of DDM, C_12_E_8_ and trans-PCC-α-M. The protein yield in the case of DDM and the purity after the two-step purification procedure^26^ was sufficient. However, the inhomogeneity of the sample as evident from the shape of the elution peak (Fig. 1a) shows that DDM does not fulfill the requirements for further, functional analysis of Pdr5. Additionally, when Pdr5 purified with DDM was assayed for ATPase activity, no activity was detected above background levels, although earlier work showed low remaining activity in DDM solubilized membrane fractions^27^. Therefore, a detergent screen was performed, using the oligomycin sensitive ATPase activity of solubilized plasma membranes containing Pdr5 as an indicator^25,27,28^ (not shown). Besides the initially promising results for DDM, C_12_E_8_ extracts showed rather high ATPase activity. Unfortunately, the following SEC showed again an inhomogeneous elution peak (Fig. 1b), which ruled out further use of this detergent.

**Figure 2:**
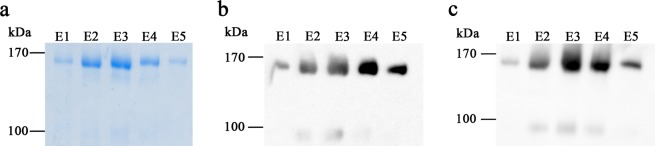
(**a**) Colloidal coomassie stained SDS-PAGE gel showing the elution fractions of the SEC following the affinity purification with trans-PCC-α-M. Western Blot of the elution fractions of the SEC with trans-PCC-α-M detecting (**b**) anti-Penta-His and (**c**) anti-Pdr5 specific antibody. Purified Pdr5 migrates at a molecular weight of approximately 170 kDa. The elution fractions E1-E5 for trans-PCC-α-M purified Pdr5 correspond to the SEC chromatograms in Fig. 1.

**Figure 3:**
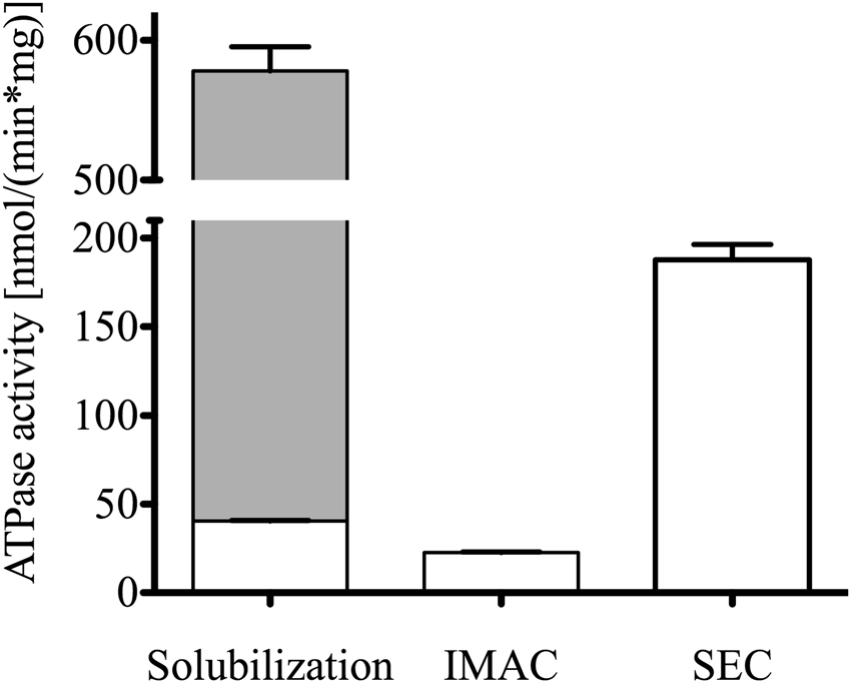
Pdr5-specific ATPase activity after each purification step. After solubilization, oligomycin-sensitive ATPase was measured (grey bar: overall activity, white bars: Pdr5-specific activity). The error bars represent the data of three independent measurements with the standard deviation reported as error.

We therefore focused on other members of the class of maltosides and an only recently commercially available member of this detergent class, trans-PCC-α-M (*trans*-4-(*trans*-4′-propylcyclohexyl)cyclohexyl-α-d-maltoside)^29^ was chosen to be tested in all purification steps. Starting with the solubilization, trans-PCC-α-M showed nearly identical results to DDM with respect to the yield after solubilization. The yield of the subsequent affinity purification was even higher as compared to DDM (up to 1 mg of protein per L cell culture compared to 0.3 mg/L cell culture). Figure 1c shows the SEC chromatograms performed with trans-PCC-α-M for wild-type Pdr5 as well as the E1036Q mutant that behaved identically during solubilization and purification. Most importantly and in clear contrast to other tested detergents, the purification protocol using trans-PCC-α-M resulted in a solubilized Pdr5 protein in a highly homogenous state, without aggregates at high purity as evident from the SDS-PAGE shown in Fig. 2a, as well as in the Western blots using anti-Penta-His (Fig. 2b) and polyclonal, anti-Pdr5 antibodies (Fig. 2c). These findings were supported by mass spectrometry (MS) analysis, which demonstrated that more than 90% of peptides observed in the MS spectra were derived from Pdr5 (not shown). Additionally, with trans-PCC-α-M, purified Pdr5 possessed a preserved ATPase active during all steps of the purification (Fig. 3). Thus, with respect to our objectives, trans-PCC-α-M turned out to be the detergent of choice.

### Pdr5 is a monomer in solution

**Figure 4:**
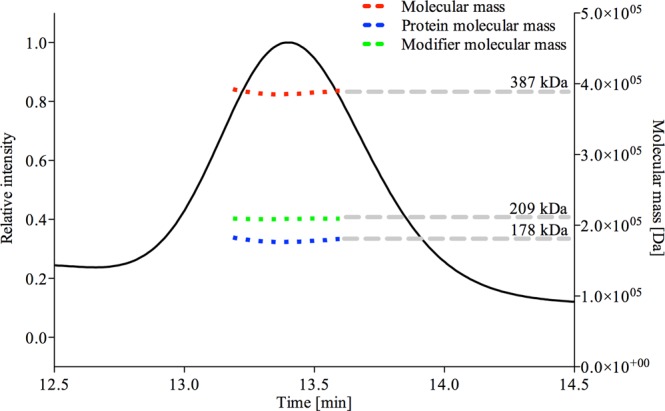
SEC-MALS analysis of purified Pdr5. The d*n*/dc value for trans-PCC-α-M was calculated in an independent measurement and used for the conjugate analysis. The overall mass of the protein-detergent complex was calculated as 387 kDa (red dashed line), with a protein molecular mass of 178.2 ± 0.2 kDa (blue dashed line). The modifier mass (green dashed line) corresponds to the detergent micelle mass.

Pdr5 is a full-size ABC transporter comprising two NBDs and two TMDs encoded on a single gene^13^. Previously, a low resolution structure (25 Å), obtained by electron microscopy, of Pdr5 in a lipid bilayer showed a square-like arrangement of particles that led to the conclusion that Pdr5 may form a dimer of two full-size transporters^26^. To assess whether Pdr5 is monomeric or forms higher oligomeric species in solution, multi-angle light scattering in combination with SEC (SEC-MALS) analysis was performed^30^. As Pdr5 is detergent solubilized, it was necessary to first determine the value of d*n*/d*c* as a measure of Pdr5 bound trans-PCC-α-M to be able to distinguish between the mass of the micelle and the protein. The d*n*/d*c* value is the specific refractive increment that corresponds to the changes in the refractive index in relation to the change in concentration of the investigated macromolecule and thereby enables to distinguish between the mass of the protein and the bound detergent^30^. Using a batch method with different detergent concentrations^30,31^ we obtained a value of d*n*/d*c* = 0.1392 ± 0.001 ml/g. As shown in Fig. 4, this resulted in a calculated protein mass of M_W_ = 178.2 ± 0.2 kDa, close to the theoretical mass of 173.1 kDa, including the histidine tag and protease cleavage site, demonstrating that Pdr5 is indeed a monomer in solution.

### NTPase activity of purified Pdr5

**Figure 5:**
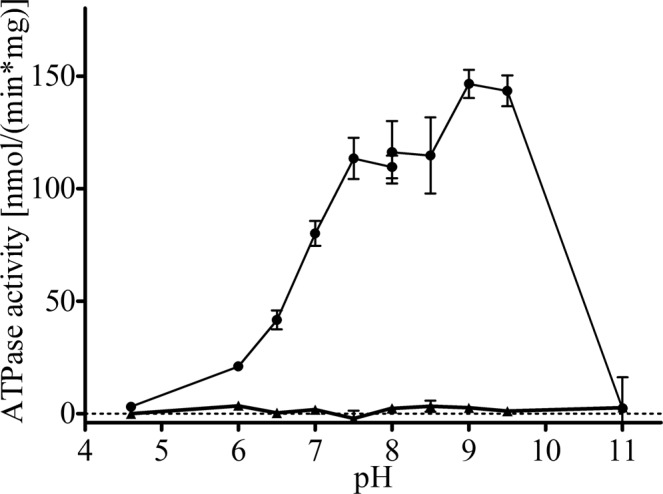
pH dependency of the ATPase activity of purified WT (•) and E1036Q (▴) Pdr5. Release of inorganic phosphate was measured after 20 min incubation with 4 mM ATP. Error bars represent three independent measurements (n = 3).

**Figure 6:**
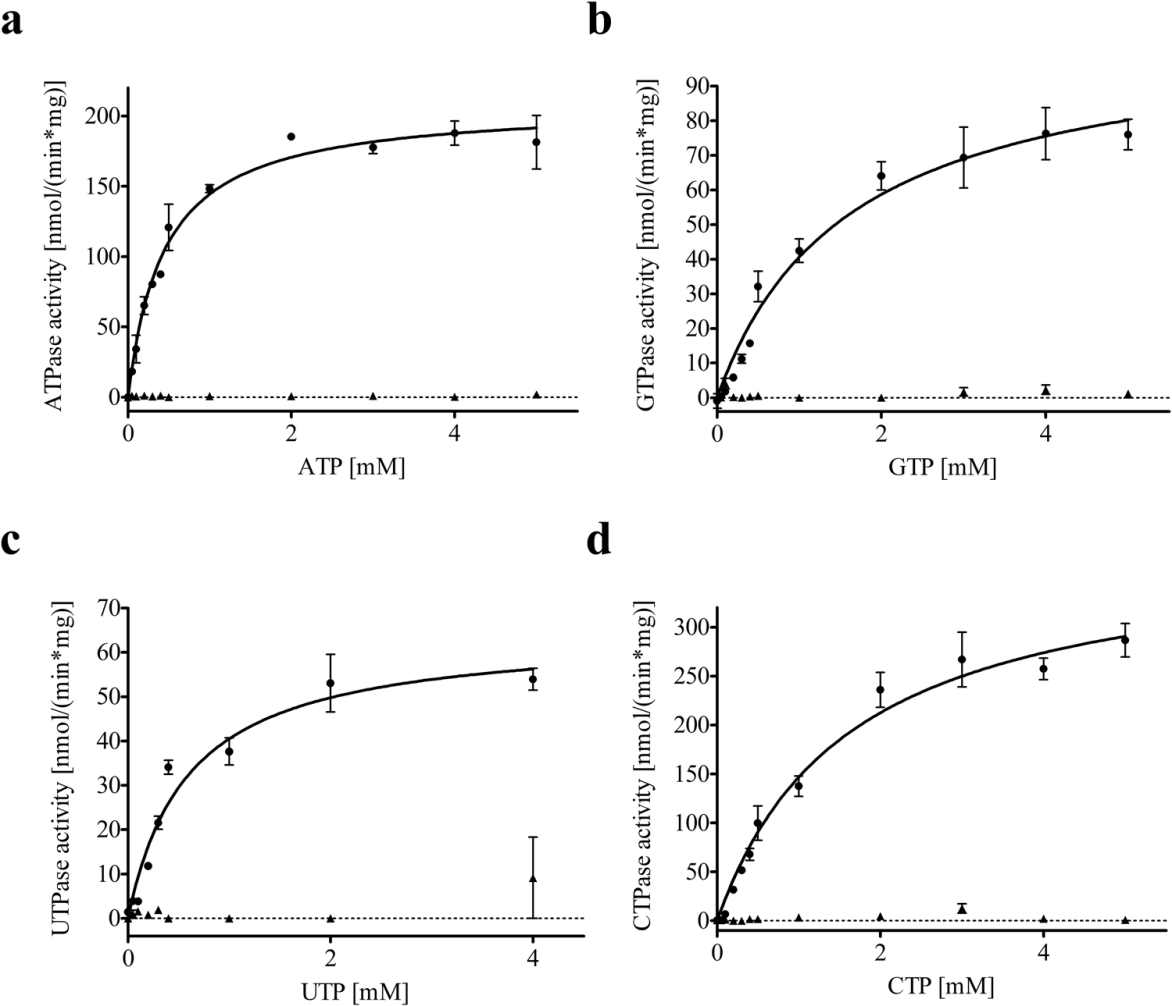
NTPase activity of purified WT (•) and E1036Q (▴) Pdr5. (**a**) ATPase activity of Pdr5. (**b**) GTPase activity of Pdr5. (**c**) UTPase activity of Pdr5. (**d**) CTPase activity of Pdr5. In all experiments 0–5 mM of NTPs were added and the release of inorganic phosphate was measured after 20 minutes. The error bars represent the data of at least three independent measurements (n = 3). Kinetic parameters of the Pdr5 specific NTPase activity can be found in Table 1.

NTPase activity of purified wild type Pdr5 was performed by a colorimetric NTPase activity assay as described in detail by Baykov *et al*.^32^. The E1036Q Pdr5 mutant, which does not exhibit any significant ATPase activity^25^, purified by the same protocol, was used as a negative control. Plasma membrane embedded Pdr5 exhibits the highest ATPase activity at pH 9.5^25^. In order to validate whether this is the case for detergent purified Pdr5, we analyzed the pH dependency of the ATPase activity of Pdr5 in detergent solution. As summarized in Fig. 5, the activity has a broad range with a maximum at pH 9.5. Figure 6 shows the NTPase activity of the WT and E1036Q Pdr5 at different NTP concentrations. At saturating substrate concentrations the measured ATPase activity of WT Pdr5 was 208.5 ± 6.3 nmol/(min*mg) with a K_m_ of 0.44 ± 0.05 mM while the E1036Q mutant did not exhibit any significant activity above background (see Figure 6a and Table 1). In the context of Golin *et al*.^33^, it was of interest to analyze the efficiency of other NTPs as it is suggested that the ABC transporter Pdr5 can utilize other nucleotides to fuel its transport activity. As summarized in Fig. 6 and Table 1, Pdr5 exhibits significant NTPase activity for all nucleotides tested, with the lowest efficiency for UTP and GTP (v_max_/K_m_ of 0.0001 L/mg/min) followed by CTP (v_max_/K_m_ of 0.0002 L/mg/min), and with the highest efficiency for ATP (v_max_/K_m_ of 0.0005 L/mg/min). Although the v_max_ of the CTPase is higher than the ATPase activity, only the latter displays a K_m_ that is in respect to NTP concentrations within the physiological range (intracellular NTP levels: ATP: 1.51 ± 0.32 mM, GTP: 0.30 ± 0.02 mM, CTP: 0.21 ± 0.03 mM, UTP: 0.33 ± 0.08 mM^34^) that allows the transporter to work always at saturating levels of nucleotide. The E1036Q mutation in the Walker B motif of NBD2 led to no significant activity in any of the performed assays (Figs 5 and 6), which demonstrates on the one hand that no detectable impurities are present and more importantly that in fact the degenerated NBS is ATPase deficient as shown earlier^25^. As proposed by Golin *et al*.^33^, we show here that GTP can be used as an energy source for Pdr5. However, with the K_m_ being above intracellular GTP levels, the significance of this nucleotide for active drug efflux under physiological conditions remains unclear.

**Table 1:** Kinetic parameters of purified Pdr5 NTPase activity.

NTP	V_max_ [nmol/mg/min]	K_m_ [mM]	V_max_/K_m_ [L/mg/min]
ATP	208.5 ± 6.3	0.44 ± 0.05	0.0005
GTP	106.0 ± 7.9	1.61 ± 0.30	0.0001
UTP	64.6 ± 4.2	0.59 ± 0.11	0.0001
CTP	384.9 ± 23.2	1.62 ± 0.25	0.0002

### Inhibitory effects of substrates and inhibitors on the ATPase activity of purified Pdr5

**Figure 7:**
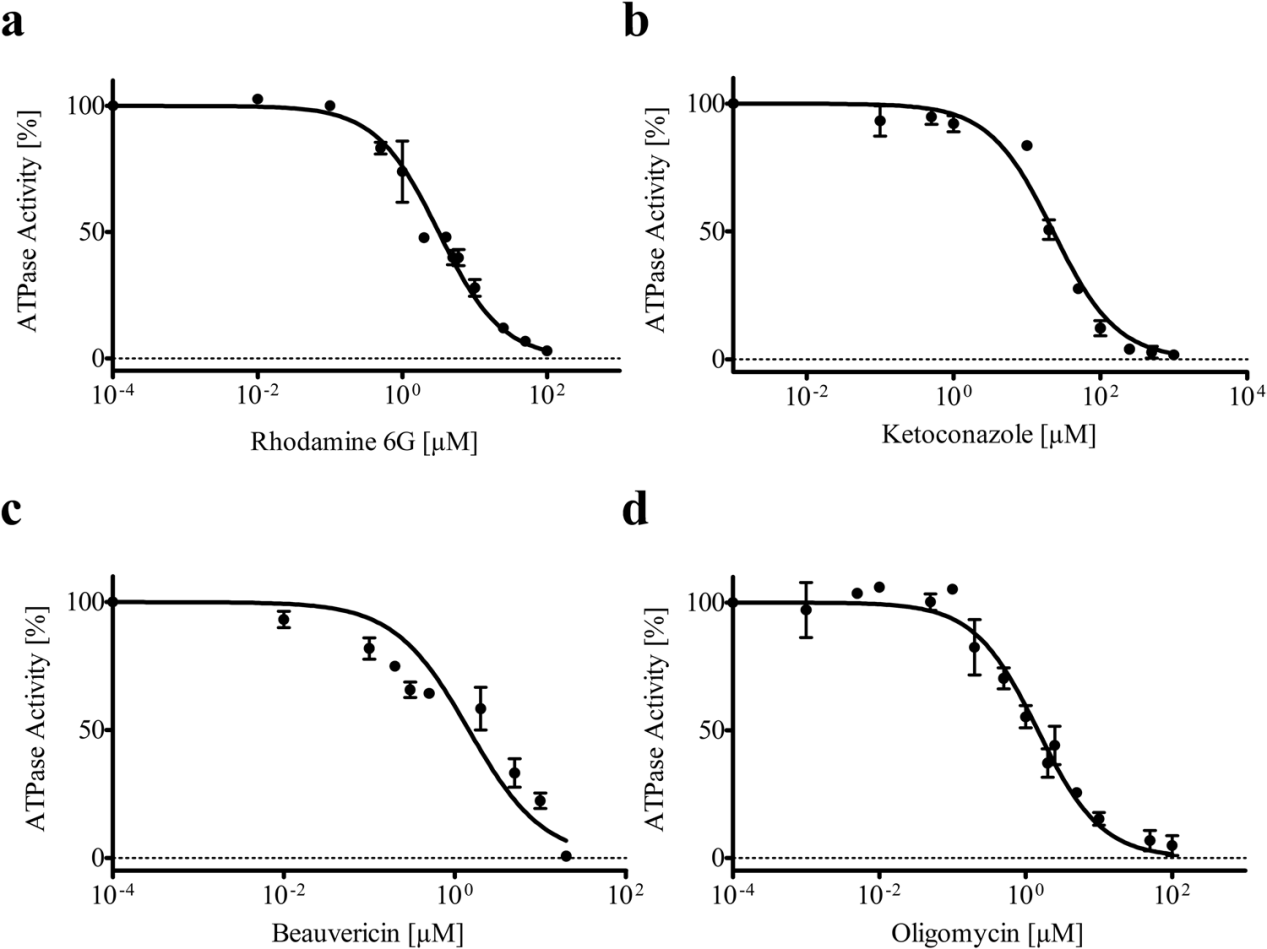
Inhibition of the ATPase activity of purified Pdr5 by its substrates and inhibitors. (**a**) rhodamine 6 G, (**b**) ketoconazole, (**c**) beauvericin, and (**d**) oligomycin. Error bars represent 3 independent measurements (n = 3).

For plasma membrane vesicles it has been previously shown that Pdr5 is a strictly uncoupled transporter^25^, as none of the known substrates of Pdr5 were able to stimulate its ATPase activity. However, some substrates as well as inhibitors are able to reduce the ATPase activity up to complete inhibition^25^. This characteristic allows to validate whether detergent solubilized and purified Pdr5 shows the same or similar characteristics as embedded in its physiological environment, the lipid bilayer. This also opens up a suitable way to compare the functional properties of the transporter in solution with the one embedded in the membrane. We therefore measured the effect of several substrates and inhibitors on its ATPase activity. As shown in Fig. 7, all tested inhibitors and substrates are able to inhibit the purified Pdr5 transporter comparable to what was observed in plasma membranes. The respective K_i_ of each effector-compound is summarized in Tables 2 and 3, respectively. Interestingly, in solution the concentration necessary to inhibit the ATPase activity of purified Pdr5 by its substrates rhodamine 6 G and ketoconazole is roughly 5 times higher than determined for Pdr5 in plasma membrane vesicles (Table 2). Moreover, for inhibitors the corresponding values are even up to 300-fold increased (Table 3). All tested Pdr5 substrates and inhibitors are hydrophobic molecules and have similar partition coefficients to ketoconazole^35^ (Tables 1 and 2), preferring the hydrophobic nature of a biological membrane. Thus, the local concentrations of the drugs in the membrane are several orders higher than in solution. Therefore, the increase in K_i_ for purified Pdr5 compared to the membrane system is not surprising. Additionally, these values may reflect the more flexible environment for the protein within a detergent micelle compared to a lipid bilayer. Other tested substrates like cycloheximide did not inhibit the ATPase activity (not shown), similar to what was observed for Pdr5 in plasma membranes^25^. Together, the results of the Pdr5-ATPase inhibition assay confirm that detergent purified Pdr5 shows comparable properties and characteristics as in plasma membranes.

**Table 2:** Substrate-mediated inhibition of Pdr5 ATPase activity and substrate partition coefficient.

	Purified Pdr5	Pdr5 in plasma membrane^a^	K_i, detergent_: K_i, PM_	Partition coefficient
Substrate	K_i_ [µM]	K_i_ [µM]		logP
Rhodamin 6 G	3.2 ± 0.2	0.6 ± 0.1	5.3	6.52
Ketoconazol	22.73 ± 2.1	5.4 ± 0.8	4.2	4.06

^a^Taken from^25^.

**Table 3:** Inhibitor-mediated inhibition of Pdr5 ATPase activity and inhibitor partition coefficient.

	Purified Pdr5	Pdr5 in plasma membrane	K_i, detergent_: K_i, PM_	Partition coefficient
Inhibitor	K_i_ [µM]	K_i_ [µM]		logP
Oligomycin	1.46 ± 0.13	0.088^b^	16.2	6.49
Beauvericin	1.45 ± 0.30	0.004 ± 0.002^c^	362.5	9.57

^b^Taken from^27^ for UTPase activity.

^c^Taken from^44^.

### The asymmetric ABC transporter Pdr5 does not exhibit adenylate kinase activity

**Figure 8:**
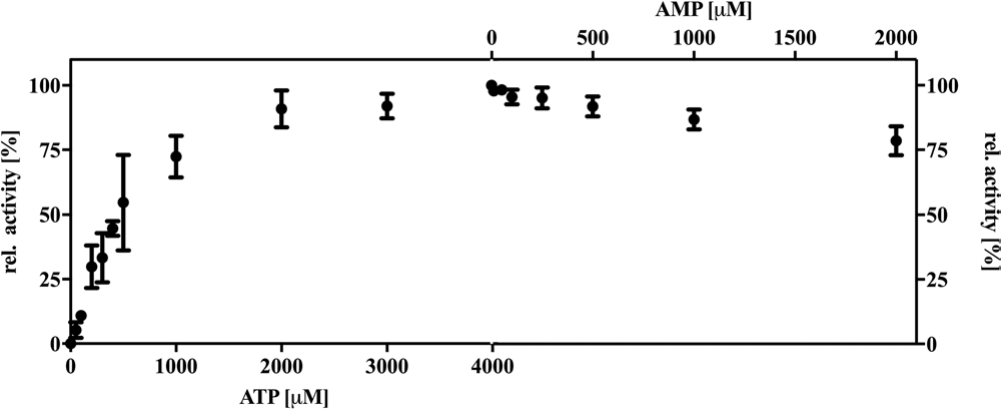
Test for adenylate kinase activity of Pdr5 as described in^37^. The relative activity in percent corresponds to the formation of ADP as a function of NADH consumption in an enzyme coupled assay. An increasing amount of AMP at saturating ATP concentration (4 mM) was added and in case of adenylate kinase activity, an increase in activity should be observerd. Error bars represent 3 independent measurements.

We tested whether Pdr5 possesses an adenylate kinase (AK) activity, as it was reported for other asymmetric ABC transporters like CFTR and TmrAB^36,37^. Therefore, the formation of ADP through the AK catalyzed reaction of *ATP* + *AMP* → 2*ADP* was assayed as a function of NADH consumption in the enzyme coupled ATPase assay. In the case of an AK activity, addition of AMP would increase the ADP concentration additionally to the formation through the ATPase activity of the protein. The method used in this study was first described for CFTR^37^. Since Pdr5 exhibits a high basal ATPase activity that is not further stimulated by its substrates, as described in this study, other methods of assaying AK activity are in our opinion unsuitable. However, as seen in Fig. 8, the addition of AMP to the reaction did not increase the formation of ADP. Conversely, it led to a slight decrease in NADH consumption, which indicates a minor inhibition of the ATPase activity of Pdr5 with a maximum inhibition of about 20% at 2 mM AMP. We therefore can rule out that Pdr5 exhibits an adenylate kinase activity that was shown for other ABC transporter^36^.

## Discussion

Pdr5 is the major ABC protein in the PDR network of *S*. *cerevisiae*^9,38^ conferring resistance to many unrelated exogenous drugs or xenobiotics^12^. Although extensively studied for more than 25 years^9^, the molecular mechanism of the efflux pump remains mostly elusive, as it was not possible to investigate it in an isolated and functional state. This is most certainly due to the fact that mainly the detergent solubilization process causes an inactivation of the protein, leading to inactive protein after purification, as demonstrated in our present study. Previously, numerous mutational studies have provided important insights in residues involved in drug binding, interdomain crosstalk as well as the molecular diode function^38–43^ of the unidirectional substrate transport of Pdr5. However in order to disclose at a molecular level how these mutations affect the different steps in the pump cycle, it is necessary to isolate the ABC transporter for these mechanistic studies in a functional state and, as a final goal, to determine the 3D-structure.

In plasma membranes vesicles, it was shown that Pdr5 exhibits a high basal ATPase activity over a broad pH range with a maximum at pH 9.5 that could not be further stimulated in the presence of substrates. However, some of its substrates inhibit the ATPase activity at high concentration, possibly due to non-competitive inhibition^25,27,33^. We tested whether purified Pdr5 shows similar behavior towards the well-studied substrates ketoconazole and rhodamine 6G and two inhibitors (oligomycin^11^ and beauvericin^44^) and could demonstrate that, indeed, the substrates and inhibitors affect the ATPase activity in the same way as it was shown for Pdr5 in plasma membranes. The measured K_i_s however differed in magnitudes, especially for the inhibitors. This finding however is not surprising, given the hydrophobic nature of the substances tested, which show a high preference for partitioning into the membrane as highlighted by the partition coefficients of these compounds. For beauvericin it was shown that only nanomolar concentrations are required to inhibit the ATPase and transport activity in plasma membranes, while in whole cell assays the concentration necessary to observe an impact on the Pdr5-mediated drug efflux was within the micromolar range^44^. This indicates that the drug cannot freely partition in the membrane presumably due to polar groups on the cell surface and/or the cell wall, which are not present in isolated plasma membranes. Therefore, higher concentrations were required to obtain inhibitory effects on Pdr5 compared to isolated plasma membranes. A similar effect is likely observed in our detergent-solubilized system, where such an increased drug accumulation within the protein-detergent micelle does not occur. Moreover, oligomycin is a known inhibitor of the Pdr5 ATPase activity^11^ as well as of ATP synthase. Here, it binds to the membrane embedded F_O_ subunit, which was shown by cryo-EM structure analysis^45^. Additionally, this effect is not specific to Pdr5. It was shown for the well-studied ABC transporter P-gp that EC_50_ values for some hydrophobic compounds like verapamil with a logP value of 4.55 are well-above 300 times higher in a detergent system compared to P-gp embedded in a lipid bilayer^46^.

Pdr5 exhibits significant NTPase activity for all NTPs as shown in this study. Although ATP shows the highest efficiency with a K_m_ below physiological ATP concentrations, we cannot rule out that the transporter might utilize other NTPs. In the light of the work of Golin *et al*.^33^, in which it was shown that substrate inhibition for GTP fueled transport takes place at higher concentrations compared to ATP mediated efflux and the ‘kinetic substrate selection model’ proposed by Ernst *et al*.^47^ it can be speculated that the NTPs are utilized depending on the present substrate and its concentration as they exhibit different kinetic parameters. Additionally, it was shown for the multidrug transporter PatA/PatB that GTP is actually the preferred nucleotide to energize the translocation of its substrates compared to ATP^48^. This indicates that the choice of nucleotide might depend on several factors and it is not necessarily ATP inducing NBD dimerization and being hydrolyzed to energize the substrate efflux of ABC transporters, as it was also proposed in a study of a NBD from *Methanocaldococcus jannaschii*^49^. It is worth mentioning that the reported basal ATPase activity of Pdr5 in plasma membranes is in some cases up to 2 µmol/min/mg^25^. However, it is important to note that the catalytic efficiency (v_max_/K_m_) of Pdr5 for ATP in detergent is 0.0005 and in the case of plasma membrane bound Pdr5 0.0008 L/mg/min (values taken from^19^) which is a factor of 1.6, as the v_max_ and K_m_ are both lower compared to plasma membrane preparations. This reflects a v_max_/K_m_ compensation. Additionally, the reported activity of Pdr5 in plasma membranes varies a lot as Golin *et al*. report an activity of about 150 nmol/mg/min^33^, which is within the range of activity reported in this study.The above described findings need further support from studies in a reconstituted system. However, it was not possible so far for us to reproducibly measure rhodamine 6G transport in Pdr5 containing proteoliposomes.

The low resolution structure of Pdr5 suggested that Pdr5 might function as a dimer in the membrane^26^. As Pdr5 is a full-size ABC transporter consisting of two NBDs and two TMDs within a single molecule^8^, these findings were quite surprising. However, as the author state in their study, dimerization was only observed upon detergent removal and no activity of the purified or reconstituted protein was reported^26^. It was shown for other ABC transporters like ABCA1 as well as the peroxisomal ABC transporters ABCD1 and ABCD2 that these proteins can actually form higher oligomeric functional units^50,51^. Nevertheless, for Pdr5 it is assumed that the functional unit is a monomer as shown for other full-size ABC transporters^52^. In our experiments, we used SEC-MALS analysis^30^ of the purified functional Pdr5 in trans-PCC-α-M micelles to gain information of the oligomeric state of the protein in solution. Size-exclusion chromatography, coupled with multiangle light scattering, is a most widely used technique for determining the absolute molecular weight distribution and averaged molecular weights of proteins and natural polymers^53^. Here, we show with *in vitro* assays that we purified Pdr5 in a functional state and by SEC-MALS analysis that the functional, purified Pdr5 is monomeric in solution.

In summary, the above described purification protocol for Pdr5 in a monomeric physiological competent, high active form will allow further detailed functional and structural investigations on the molecular steps during the transport cycles of Pdr5. Additionally, the presented data and purification approach could be extended to the Pdr5 homolog Cdr1 of the clinically important pathogen *C*. *albicans*. Initial studies on Cdr1 purified with Triton X-100 resulted in rather low activity and might be improved using our protocol^54^.

## Methods

### Growth media and chemicals

Yeast strains were cultured in YPD medium (20 g/l tryptone/peptone, 10 g/l yeast extract and 20 g/l glucose). All chemicals, if not stated otherwise, were obtained from Carl Roth or Sigma-Aldrich. trans-PCC-α-M was purchased from Glycon Biochemicals. anti-Pdr5 antibody was purchased from Davids Biotechnology, anti-Penta-His antibody was obtained from Qiagen.

### Yeast strains

In this study we used the *S*. *cerevisiae* strain YRE1001 (*MAT*a *ura3-52 trp1-1 leu2–3*,112 *his3–11*,15 *ade2-1 pdr1–3 pdr5pdr5promΔ::TRP1*). For more detailed information on the strain construction see^25^.

### Total membrane isolation and solubilization

Cells were grown at 30 °C in YPD medium. The nitrogen source was refreshed at an OD_600_ of 1.5 by addition of 10% (v/v) 5x YP (100 g/l tryptone/peptone, 50 g/l yeast extract). At an OD_600_ of 3.5 the cells were harvested at 5000 × g for 15 min (4 °C).

All steps of the membrane isolation were performed at 4 °C. Cells were resuspended in 50 mM Tris-HCl pH 8.0, 5 mM EDTA and two protease inhibitor tablets (EDTA-free, ROCHE). Lysis of the cells was performed with glass beads. The suspension was centrifuged twice at 1000 × g for 5 min (4 °C) and once at 3000 × g for 5 min (4 °C) to remove cell debris and the supernatant was centrifuged at 20,000 × g for 40 min (4 °C). The resulting pellet was resuspended in buffer A (50 mM Tris-HCl pH 7.8, 50 mM NaCl, 10% (w/v) glycerol) and adjusted to 10 mg/ml overall protein concentration. Solubilization of the membrane proteins was carried out with 1% (w/v) trans-PCC-α-M for 1 h under gentle stirring at 4 °C.

### Affinity purification and size exclusion chromatography

The immobilized metal ion affinity chromatography (IMAC) of N-terminal 14x histidine tagged Pdr5 was performed as described in^14^. In short, solubilized proteins were separated from the non-solubilized fraction by ultracentrifugation at 170,000 × g for 45 min at 4 °C. A 1 ml HiTrap Chelating column loaded with Zn^2+^ ions was equilibrated using low histidine buffer (50 mM Tris-HCl pH 7.8, 500 mM NaCl, 10% glycerol, 2.5 mM l-histidine, 0.003% (w/v) trans-PCC-α-M). Subsequently, the sample was loaded on the column, washing and elution was performed by a step gradient using low and high histidine buffer (50 mM Tris-HCl pH 7.8, 500 mM NaCl, 10% glycerol, 100 mM l-histidine, 0.003% (w/v) trans-PCC-α-M).

For size exclusion chromatography, the elution fractions were pooled and concentrated using a Vivaspin 6 (Sartorius) centrifugal concentrator (100 kDa MWCO). The size exclusion chromatography was performed on a Superdex 200 10/300 GL column (GE Healthcare) equilibrated with buffer A containing 0.003% (w/v) trans-PCC-α-M. Both purification steps were carried out on the Äkta protein purification systems (GE Healthcare).

### SEC-MALS analysis

To investigate the oligomeric state of purified Pdr5, multi-angle light scattering in combination with size exclusion chromatography (SEC-MALS) analysis was performed on an Agilent 1260 HPLC System. A triple-angle light scatter detector in combination with a differential refractive index detector (miniDAWN TREOS and Optilab rEX, respectively (Wyatt Technology Europe) were used and data analyzed with Astra 4 Software (Wyatt Technology Europe).

### NTPase activity assay

Purified Pdr5 (1 µg per well) was incubated with 10 mM MgCl_2_, 300 mM Tris-glycine buffer pH 9.5, 0.004% (w/v) trans-PCC-α-M and 0–5 mM NTP in a reaction volume of 25 µl.

pH dependency of the Pdr5 ATPase activity was measured as described in^25^. In short, 1 µg Pdr5 was incubated with 4 mM ATP, 10 mM MgCl_2_ and 0.004% (w/v) trans-PCC-α-M in 300 mM MES-Tris (pH 4.6–8.0) or 300 mM Tris-glycine (pH 8.0–11.0) buffer After incubation for 20 min at 30 °C, the reaction was stopped by adding 175 µl of ice-cold 40 mM H_2_SO_4_^32^.

Oligomycin (OM) sensitive ATPase activity of solubilized plasma membrane proteins was performed as described in^25,27,28^.

Released inorganic phosphate was determined by a colorimetric assay using Na_2_HPO_4_ as a standard^55^.

### ATPase inhibition assay

The inhibition assays were performed as the NTPase activity assays described above. The compounds were dissolved in an appropriate solvent (water, methanol or DMSO) and mixed with 750 mM Tris-glycine buffer to reach the desired stock concentrations. Purified Pdr5 was incubated with each substance for 5 min before the reaction was started by addition of 4 mM ATP. Detection of released inorganic phosphate was performed as described for the NTPase activity assay. The non-inhibited ATPase activity was set to 100%. The K_i_ was determined using Equation (1) as described in^25^.

1 $$v=100\ast \frac{1-[drug]}{{K}_{i}+[drug]}$$

Here, v corresponds to the relative ATPase activity, K_i_ to the inhibitory constant in mol/L constant and [drug] to the drug concentration in mol/L.

logP values of the tested compounds were calculated with the Chem3D program (Perkin Elmer) based on Molecular Networks’ chemoinformatics platform MOSES (https://www.mn-am.com/moses).

### Adenylate kinase activity assay

Adenylate kinase activity assay was performed as described in^37^. In brief, an enzyme coupled ATPase assay was performed in a 96-well plate at 30 °C and measured in a Tecan Infinite 200 PRO reader (Tecan). The reaction volume of 200 µl was composed of 300 mM Tris-glycine buffer pH 8, 0.004% (w/v) trans-PCC-α-M, 5 mM MgCl_2_, 4 mM PEP, 0.6 mM NADH, 3.5 µl pyruvate kinase/lactic dehydrogenase (PK/LDH) (Sigma-Aldrich). The reaction was started by the addition of 0–4 mM ATP. For detection of adenylate kinase activity, 0–2 mM AMP were added at saturating concentrations of ATP (4 mM). The absorbance of NADH was detected at 340 nm for 20 minutes. The decrease in NADH absorbance is proportional to the increase in ADP.
